# The utility of animal models in high fidelity trauma simulation

**DOI:** 10.1186/1757-7241-23-S2-O2

**Published:** 2015-09-11

**Authors:** John E McKenna, Rosel E Tallach

**Affiliations:** 1 Department of Anaesthesia, Royal London Hospital, London, UK

## Background

Simulation is a well-established method of training trauma teams. The use of animal models within simulation has been described by the military [1], but is less common in civilian practice. As part of the Queen Mary Masters of Trauma Sciences summer school, trauma simulation has been run with and without animal models.

## Method

Two courses were run incorporating animal models and two without. Feedback forms were reviewed. Free text learning points were mapped to the Crisis Resource Management framework [2] or recorded as technical or unclassified statements. Feedback was compared between courses to see if animal models altered the educational focus. The faculty experience of using animal models is discussed.

## Results

60 feedback forms were reviewed, 34 from courses with animal models and 26 from courses without. There were similar ratings of satisfaction in both groups. There were 167 learning points, 94 from courses with animal models and 73 from courses without. Both groups reported communication as the most common learning point. In the animal model group more candidates commented on fixation and attention errors. There were more technical learning points in the non-animal model group. Specific comments regarding the use of animal models were generally positive.

## Discussion

Animal models provide a method of simulating specific trauma skills (e.g. thoracotomy) in real time. They provide a good example of task focus and fixation. However animal models are expensive, alter scenario fidelity and provide an uneven experience for all candidates.
